# The Vulnerability of Chinese Theaceae Species Under Future Climate Change

**DOI:** 10.3390/biology15020151

**Published:** 2026-01-15

**Authors:** Xuzhe Zhao, Junfeng Tang, Jiang Zhu, Lan Yao, Xunru Ai, Hongxia Xu, Guofei Ma, Jun Jiang, Huiliang Yu, Zunwei Ke

**Affiliations:** 1 Hubei Key Laboratory of Biological Resources Protection and Utilization, Hubei Minzu University, Enshi 445000, China; xuzhe_zhao@126.com (X.Z.); zhujiang@hbmzu.edu.cn (J.Z.); hbmyyl@163.com (L.Y.); hbmyaxru@163.com (X.A.); 2 Administration of Shennongjia National Park, Shennongjia 442421, China; 13212767210@163.com (H.X.); mgf1967260290@163.com (G.M.); 15971855799@163.com (J.J.); snjpyuhl@163.com (H.Y.); kezunwei@hjnu.edu.cn (Z.K.); 3 Faculty of Biochemistry and Environmental Engineering, Hanjiang Normal University, Shiyan 442000, China; 4 Shiyan Key Laboratory of Biological Resources and Eco-Environmental Protection, Hanjiang Normal University, Shiyan 442000, China

**Keywords:** climate change, land use change, range shifts, modulating effect, mammals

## Abstract

Assessing the vulnerability of species to climate change is currently one of the hot issues in ecology and conservation biology. Although species sensitivity and adaptability play a crucial role in determining species vulnerability to climate change, most studies have only focused on habitat exposure, hindering a comprehensive understanding of species vulnerability to climate change and the implementation of effective conservation actions and policies. Here, we performed a comprehensive evaluation of the species sensitivity, habitat exposure and vulnerability of 122 Chinese Theaceae species and the spatial distribution patterns of their sensitivity, habitat exposure and vulnerability, as well as the effectiveness of China’s protected area network in protecting these species under future climate change. Our analyses suggest that species vulnerability was mainly determined by species sensitivity rather than habitat exposure. In addition, these species generally exhibit a high sensitivity and vulnerability to temperature-related variables, such as the annual mean temperature and temperature annual range, while exhibiting a high exposure to precipitation variables, such as total annual precipitation and precipitation seasonality. Furthermore, our analyses show that the high-vulnerability areas are mainly distributed in western and eastern China. However, no more than 17% of the high-vulnerability areas would be covered by China’s protected area network and no more than 15% of the median- and low-vulnerability areas would be covered by China’s protected area network. These findings can contribute to a new understanding of the vulnerability of the 122 Chinese Theaceae species to future climate change and guide effective conservation prioritizing in a rapidly changing climate.

## Simple Summary

Understanding and predicting the vulnerability of species to future climate change with their spatial distribution is crucial for biodiversity conservation. In this study, we assessed the vulnerability of 122 Chinese Theaceae species under future climate change, as well as their spatial distribution patterns, using multifaceted analyses by integrating species sensitivity and habitat exposure rather than only focusing on habitat exposure. As expected, we found that (1) species vulnerability was mainly determined by species sensitivity rather than habitat exposure; (2) these species exhibit a high sensitivity and vulnerability to temperature-related variables, while exhibiting a high exposure to precipitation-related variables; and (3) high-vulnerability areas mainly distributed in western and eastern China. However, China’s protected area network covers no more than 17% of the high-vulnerability areas and no more than 15% of the median- and low-vulnerability areas, respectively. These findings should inform a more useful dialogue determining the vulnerability of 122 Chinese Theaceae species and consequently guiding effective prioritizing conservation to offset the negative effects of future climate change.

## 1. Introduction

Safeguarding species against climate change-related risks is currently a major global priority [1,2]. However, climate change-induced local extinctions and range shifts in species are still ongoing [3,4], although numerous conservation actions and policies aimed at addressing these risks have been adopted [5]. These realities suggest that the relevant conservation actions and policies should be refined to effectively avoid the further loss of biodiversity [6,7,8]. One of the most effective paths to achieving this goal is to implement global priority conservation policies so that a large number of conservation efforts can be focused on those species and/or areas with the highest climate change vulnerability but with important conservation value [9,10]. Therefore, scientifically sound predictions of which species and/or areas will most likely be at risk under future climate change are crucial for implementing global priority conservation policies.

Currently, assessments of the vulnerability of species and/or areas to future climate change primarily rely on the application of niche-based species distribution models [11]. Such models relate current climate conditions to existing species occurrence records and project the potential species distributions for the current and future periods, thus only focusing on habitat exposure to climate change [12,13,14]. However, as outlined by previous studies [15,16,17], the degree of the vulnerability of a species to climate change not only depends on the species’ habitat exposure, but also depends on the species’ sensitivity (i.e., the ability to tolerate climate change) and the species’ adaptability (i.e., the ability to adjust to these changes). At the same time, there is a growing consensus that only considering habitat exposure and neglecting sensitivity and adaptability may hinder our full understanding of the vulnerability of species to future climate change [18]. Therefore, species’ sensitivity and adaptability must be included alongside habitat exposure when assessing species’ vulnerability to climate change and/or implementing effective conservation actions and policies. Recently, a multifaceted vulnerability analysis framework, that is, the climate niche factor analysis (CNFA), was developed by Rinnan and Lawler [19]. This framework not only can quantify species’ vulnerability by integrating species’ sensitivity and habitat exposure, but also can project the spatially explicit vulnerability of a species and thus can contribute to identifying the areas where species are expected to have the highest vulnerability to future climate change [19]. Consequently, this type of integrated approach has been widely used to estimate species’ vulnerability to climate change for many taxonomic groups, such as plants [13,20,21,22,23], fishes [18] and mammals [14,16,24].

Climate change vulnerability assessments are particularly crucial for species with important ecological and economic values [25], as it is often suggested that these types of species might usually have a higher conservation value and therefore have a higher priority for conservation compared to other species [26]. In China, Theaceae is such an ecologically and economically important angiosperm family, including about 12 genera and 274 species [27]. Although only about 17% of Chinese Theaceae species are currently threatened according to the IUCN Red List [28], many studies suggest that future climate change might exacerbate the extinction risks for threatened species and could also pose a threat to the non-threatened species, challenging the current conservation actions and policies aimed at addressing these risks in China [27,29,30]. However, previous studies have mainly focused on the climate-induced habitat exposure (i.e., distributional changes) for Chinese Theaceae species; the climate change sensitivity and vulnerability of these Theaceae species have not yet been evaluated. Moreover, although a relatively mature network of more than 2700 protected areas has been established in the past decades in China, whether this network can effectively protect Chinese Theaceae species still remains unclear. In particular, whether this network can fully cover all the areas where Chinese Theaceae species will most likely have the highest vulnerability under future climate change needs to be examined.

To fill these gaps, in this study, we aim to (1) assess the sensitivity, habitat exposure and vulnerability of each Theaceae species to future climate change; (2) map species’ spatial sensitivity under climatic conditions and map species’ spatial habitat exposure and spatial vulnerability under future climatic conditions; (3) identify priority protected areas in which species might have the highest vulnerability under future climatic conditions and assess the effectiveness of the current protected area network in protecting these high-vulnerability areas. To do so, we adopted the recently proposed CNFA framework to calculate species sensitivity, habitat exposure and vulnerability based on species occurrence records and bioclimatic variables and project their spatial distribution patterns. Then, we identified the priority protected areas according to the spatial patterns of vulnerability and performed a conservation gap analysis to assess the effectiveness of the current protected area network. These results can provide new insights in understanding species’ vulnerability under future climate change and help guide effective conservation actions and policies to safeguard species against climate change-related risks.

## 2. Materials and Methods

### 2.1. Study Area and Species Occurrence Data

This study was conducted in China, where about 46% (274 species) of the total Theaceae species worldwide are distributed [27]. The occurrence records of Chinese Theaceae species were gathered from the literature [27] and online databases [28], with the original species occurrence data collected from Chinese Virtual Herbarium (http://www.cvh.ac.cn/), the National Specimen Information Infrastructure (http://www.nsii.org.cn/) and the IUCN [28]. Overall, we obtained 15,597 occurrence records for 228 Theaceae species in China. For assessing species’ sensitivity, exposure and vulnerability and avoiding potential sampling bias, these occurrence records were overlaid onto 10 km × 10 km grid cells, and for each species only one occurrence record was retained within each grid cell. We selected this spatial resolution because 10 km × 10 km performed well in previous analyses assessing the impacts of climate change on the future distribution of Chinese Theaceae species [29]. Furthermore, as the low number of occurrence records of a species may affect the robustness of our results, following Wang et al. [13], species with <20 occurrence records were excluded in the subsequent analysis. Finally, we assessed the sensitivity, exposure and vulnerability of 122 Chinese Theaceae species with 12,851 occurrence records under future climate change.

### 2.2. Current and Future Climate Variables

To assess the sensitivity of the 122 Chinese Theaceae species, we downloaded four bioclimatic variables that are the average for the period 1970–2000 from the WorldClim dataset [resolution = 2.5 arc-minutes]; [31]: mean annual temperature (BIO1), temperature annual range (BIO7), total annual precipitation (BIO12) and precipitation seasonality (BIO15). These bioclimatic variables were used because of their ecological relevance to Theaceae species and their low multicollinearity, with Pearson’s correlation coefficients |*r*| < 0.7 and with variance inflation factor VIFs  <  5 [29]. To assess the habitat exposure and vulnerability of the 122 Chinese Theaceae species under future climate change, we also downloaded the same four bioclimatic variables from the WorldClim dataset (resolution = 2.5 arc-minutes). Specifically, for the future period, the five climate variables are the average for 2061–2080 (2070s) under two representative concentration pathways: (1) RCP2.6, which represents a low CO_2_ emission scenario, with the radiative forcing level reaching 3.1 W/m^2^ by mid-century but returning to 2.6 W/m^2^ by 2100; and (2) RCP8.5, which represents a high CO_2_ emission scenario, with the radiative forcing level reaching 8.5 W/m^2^ by 2100. In particular, these future bioclimatic variables were extracted from the global circulation model, MRI-ESM2-0, which has been recommended for use in China [32]. Finally, all four bioclimatic variables under current and future periods were resampled at a 10 km resolution using a bilinear interpolation.

### 2.3. Climate Change Vulnerability Assessments

To assess the climate change vulnerability of 122 Theaceae species in China, we first adopted a climate niche factor analysis (CNFA) developed by Rinnan and Lawler [19] to calculate the sensitivity and exposure for each species. In this analysis framework, species sensitivity was quantified based on two aspects of a species’ niche: (1) the marginality, which reflects the niche centroid distance of current climatic conditions between a species’ habitat and the whole study area; and (2) the specialization, which reflects the ratio of the size of the species’ climatic niche in the current range to that in the whole study area [33]. Accordingly, species sensitivity reflects the degree to which the ability of one species to persist at its current range is determined by the climatic conditions of its habitat. That is, the more a species is constrained by its current climatic niche quantified in its current range, the more sensitive it is to future climate change [19]. The marginality, specialization and sensitivity for each species were calculated based on species occurrence records and current climate conditions, using the ‘cnfa’ function in the CENFA package V1.1.1 [19]. Habitat exposure was quantified through a dissimilarity measure of the current and future climatic conditions within its current range. Accordingly, habitat exposure reflects the extent to which climate change will take place across the current range of a species and therefore can be calculated as a dissimilarity measure between current and future climate conditions within its current range. That is, the higher such a dissimilarity, the larger the departure of current climate conditions from future climatic conditions in its current range. The habitat exposure under RCP2.6 and RCP 8.5 scenarios by the 2070s for each species was calculated based on species occurrence records and the current and corresponding future climate conditions using the ‘departure’ function in the CENFA package V1.1.1 [19]. Then, the vulnerability for each species was quantified as the geometric mean of sensitivity and habitat exposure using the ‘vulnerability’ function in the CENFA package V1.1.1 [19].

### 2.4. Spatial Sensitivity, Exposure and Vulnerability Analysis

To investigate the spatial sensitivity, exposure and vulnerability of the 122 Theaceae species in China, we used the ‘predict’ function in the CENFA package V1.1.1 [19] to obtain the spatial patterns of sensitivity, exposure and vulnerability for each species and summarized the spatial sensitivity, exposure and vulnerability of the 122 Chinese Theaceae species in 10 km × 10 km grid cells using an assemblage-based approach following Wang et al. [13]. Specifically, we calculated the mean and standard deviation (SD) of sensitivity, exposure and vulnerability across the 122 Chinese Theaceae species for each 10 km × 10 km grid cell.

### 2.5. Identification of Priority Protected Areas and Gap Analysis

To identify the priority areas for protecting the 122 Chinese Theaceae species under future climate change, following Wang et al. [13], we first calculated the 1/3 and 2/3 quantiles for the mean spatial vulnerability across the whole study area under RCP2.6 and RCP8.5 scenarios by the 2070s, respectively. Then, we classified the mean spatial vulnerability in each grid cell into three levels, ‘High’, ’Median’ and ‘Low’, based on the above two quantiles. Specifically, the mean spatial vulnerabilities that were less than the 1/3 quantile of the mean spatial vulnerability in the whole study area were considered as ‘Low’, the mean spatial vulnerabilities that were between the 1/3 and 2/3 quantiles were considered as ‘Median’, and the mean spatial vulnerabilities that were greater than the 2/3 quantiles were considered as ‘High’. Accordingly, the areas with ‘High’ vulnerability had the highest priority for protection. To assess the effectiveness of the current nature reserves in protecting areas with different levels of vulnerability to future climate change, we also performed a gap analysis to assess the percentages of the areas in each vulnerability category within the current nature reserve network in China.

## 3. Results

### 3.1. Overall Sensitivity, Exposure and Vulnerability Across Species

**Figure 1:**
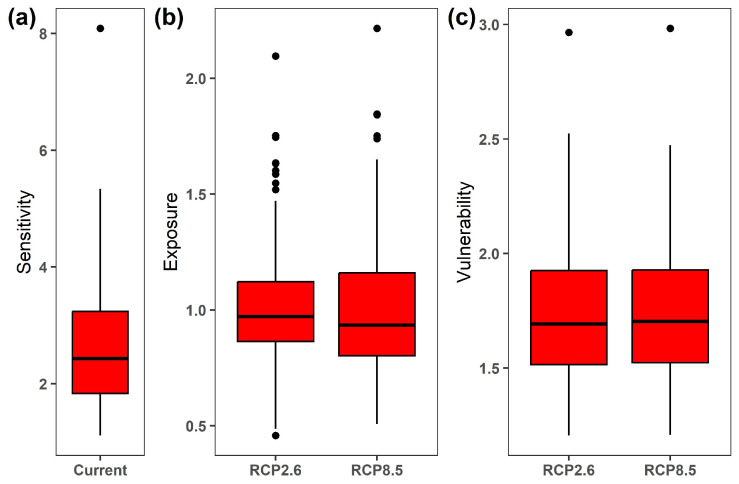
The overall sensitivity under the current period (**a**), and exposure (**b**) and vulnerability (**c**) of the 122 Chinese Theaceae species under RCP2.6 and RCP8.5 by the 2070s, respectively.

The overall sensitivity of the 122 Chinese Theaceae species ranged from 1.111 for the *Camellia fraterna* to 8.088 for the *Camellia parvimuricata* (mean sensitivity across 122 species, 2.616 ± 1.037; Figure 1a; Table S1). On the contrary, these species exhibited more similar patterns in their exposure to future climate change when compared to their sensitivity (Figure 1b). Specifically, the overall habitat exposure of the 122 Chinese Theaceae species ranged from 0.457 for the *Camellia parvimuricata* to 2.096 for the *Eurya emarginata* (mean ± SD: 1.019 ± 0.259) under the RCP2.6 scenario by the 2070s. As expected, the habitat exposure of the 122 Chinese Theaceae species under the RCP8.5 scenario (mean ± SD: 1.023 ± 0.304) was higher than that under the RCP2.6 scenario, despite having a similar pattern across the 122 Chinese Theaceae species. As a result, the *Camellia parvimuricata* had the highest overall vulnerability among the 122 species under both RCP2.6 and RCP8.5 by the 2070s, followed by *Camellia crassicolumna*, *Camellia tsingpienensis* and *Eurya hupehensis*, while *Camellia fraterna* had the lowest overall vulnerability (Table S1).

**Figure 2:**
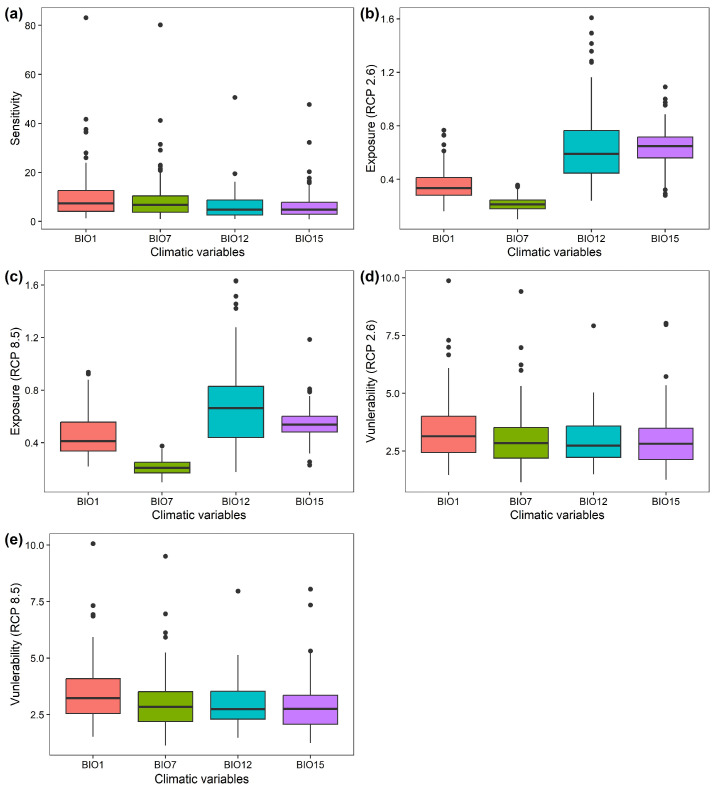
The sensitivity factors under the current period (**a**); the departure factors (**b**,**c**) and vulnerability factors (**d**,**e**) of the 122 Chinese Theaceae species under RCP2.6 and RCP8.5 by the 2070s, respectively. BIO1: mean annual temperature; BIO7: temperature annual range; BIO12: total annual precipitation; BIO15: precipitation seasonality.

Of the 122 Chinese Theaceae species, 86 species (70.49% of the 122 species) exhibited a high sensitivity to annual mean temperature, followed by temperature annual range (31 species; 25.41% of the 122 species), while only 3 (2.46% of the 122 species) and 2 species (1.64% of the 122 species) exhibit a high sensitivity to total annual precipitation and precipitation seasonality, respectively (Figure 2a; Table S2). Moreover, 63 species (51.64% of the 122 species) and 59 species (48.36% of the 122 species) exhibit a high departure to the annual mean temperature, total annual precipitation and precipitation seasonality under RCP2.6, respectively (Figure 2b; Table S3). Similarly, 79 species (64.75% of the 122 species) and 43 species (35.25% of the 122 species) exhibit a high departure to total annual precipitation and precipitation seasonality under RCP8.5, respectively (Figure 2c; Table S4). In particular, no species exhibits a high departure to the annual mean temperature and temperature annual range (Figure 2c; Table S4). As a result, the 122 Chinese Theaceae species generally exhibit a high overall vulnerability to the annual mean temperature (73 species under RCP2.6 and 94 species under RCP8.5) and temperature annual range (15 species under RCP2.6 and 10 species under RCP8.5) under both RCP2.6 and RCP8.5 by the 2070s (Figure 2d,e; Tables S5 and S6).

### 3.2. Spatial Patterns of Sensitivity, Exposure and Vulnerability

**Figure 3:**
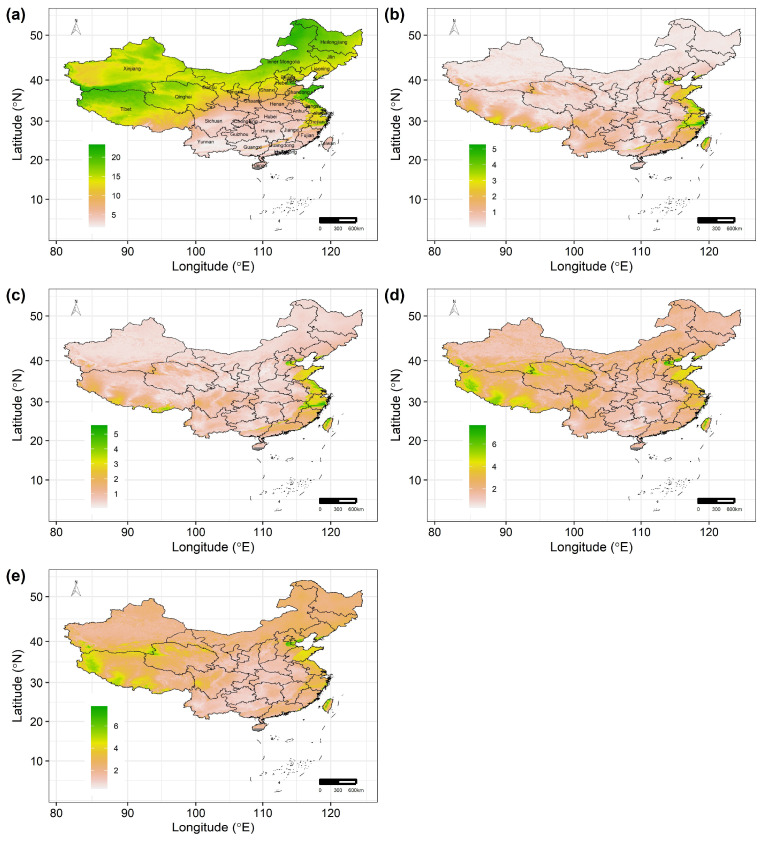
The average values of predicted sensitivity (**a**), exposure under RCP2.6 (**b**) and RCP8.5 (**c**) and vulnerability under RCP2.6 (**d**) and RCP8.5 (**e**) by the 2070s. The black lines in (**a**–**e**) are the boundaries of the provinces in China.

The mean spatial sensitivity of the 122 Chinese Theaceae species across the whole study area ranged from 1.736 to 23.244 (the average of mean spatial sensitivity, 12.015 ± 5.418; Figure 3a). Most areas of the Heilongjiang, Jilin, Inner Mongolia, Tianjin and Shandong provinces are more sensitive to future climate change for these species than other regions. In addition, the 122 Chinese Theaceae species exhibit a similar exposure and vulnerability to future climate change because of the low standard deviation of the mean spatial exposure and vulnerability. In particular, the spatial exposure and vulnerability for these Chinese Theaceae species are higher under RCP8.5 than under RCP2.6. Specifically, the mean spatial exposure of the 122 Chinese Theaceae species across the whole study area ranged from 0.022 to 5.274 (the average of mean spatial exposure, 0.648 ± 0.659) under RCP2.6 (Figure 3b) and from 0.038 to 5.586 (the average of mean spatial exposure, 0.777 ± 0.648; Figure 3c) under RCP85. Similarly, the mean spatial vulnerability of the 122 Chinese Theaceae species across the whole study area ranged from 0.285 to 7.667 (the average of mean spatial exposure, 2.045 ± 0.873) under RCP2.6 (Figure 3d) and from 0.320 to 7.765 (the average of mean spatial exposure, 2.298 ± 0.910; Figure 3e) under RCP8.5. Moreover, these Theaceae species will experience a high exposure in some regions of the Tibet, Jiangsu, Zhejiang and Taiwan provinces (Figure 3b,c), while these Theaceae species will experience a high vulnerability in Tianjin city, the western part of Tibet and the middle of the Taiwan province (Figure 3d,e).

### 3.3. Priority Protected Areas and Conservation Gap Analysis

**Figure 4:**
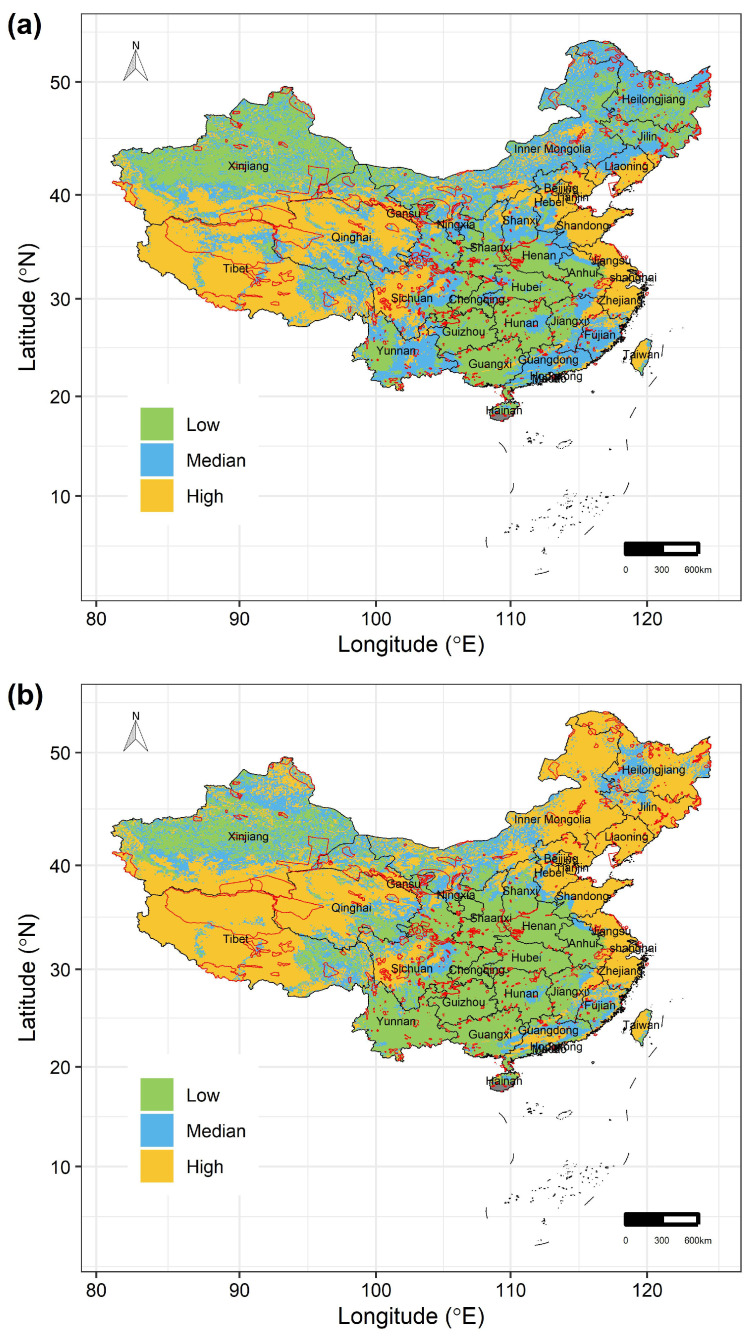
The spatial distribution of the three levels of vulnerability of the 122 Chinese Theaceae species in the whole study area under (**a**) RCP2.6 and (**b**) RCP8.5 by the 2070s. The black lines in (**a**,**b**) are the boundaries of the provinces in China. The red lines in (**a**,**b**) are the boundaries of nature reserves in China.

Under RCP 2.6 scenarios, by the 2070s, the areas where the 122 Chinese Theaceae species are at ‘Low’ vulnerability mainly occur in the Gansu, Shaanxi, Hubei, Hunan, Anhui, Guizhou, Jiangxi and Guangxi provinces, while the areas where the species are at ‘High’ vulnerability mainly occur in the western and eastern parts of China (Figure 4a). Particularly, these species are expected to face greater climate-related risks in the Tibet, Qinghai, Sichuan, Liaoning, Tianjin, Shandong, Jiangsu, Shanghai, Zhejiang and Taiwan provinces, for the percentage of the area of ‘High’ vulnerability in these regions would be up to 50% of their total areas, respectively (Figure 4a). Moreover, the areas with ‘High’ vulnerability would be larger under RCP8.5 than under RCP2.6 scenarios (Figure 4b). Specifically, these species are also expected to face greater climate-related risks in Heilongjiang, Jilin and Inner Mongolia under RCP8.5 scenarios by the 2070s. However, our conservation gap analyses show that only 17.142% and 15.961% of these high-vulnerability areas under RCP2.6 and RCP8.5 scenarios by the 2070s, respectively, would be covered by the current PAs (Figure 4). Moreover, although the 122 Chinese Theaceae species are mainly distributed in the areas of ‘Low’ and ‘Median’ vulnerability, the current PA network covers < 15% of these areas (Figure 4).

## 4. Discussion

The climate change-induced risks for species continue to attract attention, but the local extinctions and range shifts in species are still continuing. The problem derives to some degree from misleading conservation actions and policies, which were guided by climate change vulnerability assessments which only focused on habitat exposure and ignored species’ sensitivity and vulnerability. In this study, we have tried to circumvent these issues by performing a comprehensive evaluation of the species sensitivity, habitat exposure and vulnerability of 122 Chinese Theaceae species and their spatial distribution patterns, as well as the effectiveness of China’s protected area network in protecting these species under future climate change. Our analyses suggest that species vulnerability was mainly determined by species sensitivity rather than habitat exposure, and these species generally exhibit a high vulnerability to temperature-related variables, such as the annual mean temperature and temperature annual range. Moreover, our analyses show that the high-vulnerability areas are mainly distributed in western and eastern China. However, the current protected area network covers <17% of the high-vulnerability areas and covers <15% of the median- and low-vulnerability areas, respectively. These findings can contribute to a new understanding of the status, trends and threats for 122 Chinese Theaceae species by identifying risks and prioritizing conservation in a rapidly changing climate.

Habitat exposure has been evaluated extensively and used as a surrogate for species vulnerability in many previous studies [12,13,14]. However, our results suggest that species sensitivity rather than habitat exposure plays an important role in determining species vulnerability. For example, *Eurya emarginata* was ranked as the highest exposure species under all future scenarios, but its vulnerability ranked 89th among the 122 species due to its low sensitivity (ranked 97th among all species). On the contrary, although *Camellia parvimuricata* had the lowest habitat exposure among all species, it had the highest vulnerability under all future scenarios because of its highest sensitivity. Consistent with these previous studies, these findings indicate that only considering habitat exposure cannot accurately represent the vulnerability of a species to future climate change and accordingly can lead to misleading conservation actions and policies [18]. Therefore, these findings also highlight the importance of using multifaceted analyses by integrating species sensitivity and habitat exposure to assess species vulnerability to better inform conservation actions and policies.

Moreover, our results suggested that the high-vulnerability areas for the 122 Chinese Theaceae species are mainly distributed in the eastern and western parts of China under all climate change scenarios. One possible reason of these patterns is that most of the Chinese Theaceae species are mainly distributed in southern China’s tropical and subtropical moist lowlands rather than in the eastern and western parts of China [28,34]. In these regions, Chinese Theaceae species are extremely vulnerable to climate change due to their narrow suitable climate conditions and very small populations [26]. As a result, the emergence of novel climates in these regions under future periods may potentially lead to the rapid local extinction of species. These findings parallel previous studies, which also found the particularly high vulnerability of Chinese Theaceae species to future climate change in these regions [29,30].

Similarly, our results also suggested that the priority conservation areas for protecting Chinese Theaceae species are also mainly distributed in the western and eastern parts of China, such as the Tibet, Qinghai, Sichuan, Liaoning, Tianjin, Shandong, Jiangsu, Shanghai, Zhejiang and Taiwan provinces. However, we found that the identified priority conservation areas for Chinese Theaceae species are highly fragmented. Moreover, our results suggest that there will be a large conservation gap within the current protected area network in China, as this network will miss a large proportion of high-vulnerability areas. Specifically, no more than 17% of the high-vulnerability areas are covered by the current protected area network in China, and, worse still, no more than 15% of the median- and low-vulnerability areas in which the Chinese Theaceae species are mainly distributed are covered by the current protected area network in China. The habitat fragmentation of the priority conservation areas for Chinese Theaceae species, together with their large conservation gaps, poses a grave challenge for the conservation of Chinese Theaceae species under future climate change. Therefore, implementing effective conservation actions and policies, such as by expanding the current protected area network in China, can restore ecosystems, increase habitat connectivity and conserve the Chinese Theaceae species [5].

Despite our study having circumvented some issues in previous studies, our analysis framework still has some shortcomings. First, like the climate niche factor analysis framework, species’ adaptability to climate change was not included in our framework. This is largely because the data that are used to assess the ability of a species to adjust to climate change are not available for many of the 122 Chinese Theaceae species. Therefore, additional studies are required to complement our findings by incorporating species-specific dispersal ability and species’ evolutionary history into our analysis framework. Moreover, due to a potential sampling bias, the detection of these Chinese Theaceae species might be potentially imperfect [35], which also potentially has a great effect on the measurement of species sensitivity and habitat exposure. Therefore, future research should also take into account how the imperfect detection of species might impact the assessment of species’ sensitivity, habitat exposure and vulnerability.

## 5. Conclusions

In conclusion, we assessed the species’ sensitivity, habitat exposure and vulnerability of 122 Chinese Theaceae species and their spatial distribution patterns, as well as the effectiveness of China’s protected area network in protecting these species under future climate change. Notably, the vulnerability of 122 Chinese Theaceae species to future climate change was mainly determined by their sensitivity rather than habitat exposure, and they generally exhibit a high vulnerability to temperature-related variables, such as the annual mean temperature and temperature annual range. Moreover, the high-vulnerability areas for these Theaceae species are mainly distributed in western and eastern parts of China due to the narrow suitable climate conditions and small populations in these regions. However, there will be a large conservation gap within the current protected area network; that is, this network covers <17% of the high-vulnerability areas and covers <15% of the median- and low-vulnerability areas, respectively. By explicitly incorporating species sensitivity and habitat exposure to assess species vulnerability, we demonstrate the potential importance of using measures of niche width to assess species’ vulnerability and help identify promising conservation actions and policies that can form the basis for prioritizing conservation in a rapidly changing climate.

## Data Availability

The original contributions presented in the study are included in the article; further inquiries can be directed to the corresponding author.

## Supplementary Materials

The following supporting information can be downloaded at: https://www.mdpi.com/article/10.3390/biology15020151/s1, Table S1: Overall sensitivity, exposure and vulnerability of the 122 Chinese Theaceae species under RCPs 2.6 and 8.5 by the 2070s, respectively; Table S2: The sensitivity factor of the 122 Chinese Theaceae species for four bioclimatic variables; Table S3: The exposure factor of the 122 Chinese Theaceae species for four bioclimatic variables under RCP 2.6 scenario by the 2070s; Table S4: The exposure factor of the 122 Chinese Theaceae species for four bioclimatic variables under RCP 8.5 scenario by the 2070s; Table S5: The vulnerability factor of the 122 Chinese Theaceae species for four bioclimatic variables under RCP 2.6 scenario by the 2070s; Table S6: The vulnerability factor of the 122 Chinese Theaceae species for four bioclimatic variables under RCP 8.5 scenario by the 2070s.
