# Supplementary material for: The Vulnerability of Chinese Theaceae Species Under Future Climate Change
Source: Biology (Basel). 2026 Jan 15;15(2):151. doi: 10.3390/biology15020151 (PMC12837319; doi:10.3390/biology15020151)
Supplement: Supplementary file 1 [file biology-15-00151-s001.zip › Table S1. Overall sensitivity, exposure and vulnerability of the 122 Chinese Theaceae species under RCPs 2.6 and 8.5 by the 2070s, respectively..pdf]

**Table S1.** Overall sensitivity, exposure and vulnerability of the 122 Chinese Theaceae species under RCPs 2.6 and 8.5 by the 2070s, respectively.

| Species | Sensitivity | Exposure | | Vulnerability | |
|---|---|---|---|---|---|
| | | RCP 2.6 | RCP 8.5 | RCP 2.6 | RCP 8.5 |
| Camellia caudata | 2.139 | 1.059 | 1.163 | 1.596 | 1.607 |
| Schima parviflora | 1.625 | 1.123 | 1.225 | 1.41 | 1.423 |
| Schima superba | 1.707 | 1.471 | 1.588 | 1.471 | 1.483 |
| Camellia furfuracea | 2.049 | 1.194 | 1.304 | 1.572 | 1.584 |
| Schima remotiserrata | 2.209 | 1.042 | 1.162 | 1.625 | 1.642 |
| Adinandra hainanensis | 3.619 | 0.714 | 0.92 | 2.007 | 2.029 |
| Camellia japonica | 1.653 | 1.432 | 1.536 | 1.448 | 1.458 |
| Camellia oleifera | 1.643 | 1.075 | 1.119 | 1.408 | 1.416 |
| Camellia sinensis | 1.618 | 1.044 | 1.078 | 1.392 | 1.399 |
| Eurya chinensis | 1.927 | 1.141 | 1.267 | 1.536 | 1.549 |
| Eurya ciliata | 2.968 | 0.819 | 0.952 | 1.857 | 1.87 |
| Eurya nitida | 1.676 | 1.003 | 1.043 | 1.414 | 1.422 |
| Eurya trichocarpa | 2.787 | 0.817 | 0.932 | 1.791 | 1.806 |
| Polyspora axillaris | 2.542 | 1.746 | 1.843 | 1.787 | 1.789 |
| Schima crenata | 1.65 | 0.978 | 0.987 | 1.398 | 1.4 |
| Ternstroemia kwangtungensis | 1.76 | 1.021 | 1.138 | 1.45 | 1.461 |
| Ternstroemia microphylla | 3.485 | 0.701 | 0.898 | 1.955 | 1.975 |
| Pyrenaria microcarpa | 1.828 | 1.753 | 1.846 | 1.539 | 1.549 |
| Anneslea fragrans | 2.267 | 1.012 | 0.969 | 1.646 | 1.642 |
| Cleyera japonica | 1.348 | 1.158 | 1.212 | 1.285 | 1.293 |
| Eurya groffii | 2.357 | 0.965 | 0.974 | 1.671 | 1.675 |
| Eurya japonica | 1.348 | 1.419 | 1.448 | 1.31 | 1.315 |
| Eurya loquaiana | 1.62 | 1.032 | 1.037 | 1.398 | 1.402 |
| Ternstroemia gymnanthera | 1.654 | 1.051 | 1.019 | 1.412 | 1.412 |
| Ternstroemia luteoflora | 1.991 | 1.004 | 1.045 | 1.545 | 1.552 |
| Camellia fluviatilis | 3.24 | 0.718 | 0.897 | 1.905 | 1.927 |

| | | | | | |
|---|---|---|---|---|---|
| Camellia kissii | 2.693 | 0.886 | 0.995 | 1.774 | 1.787 |
| Eurya acutisepala | 2.339 | 0.815 | 0.833 | 1.647 | 1.654 |
| Eurya muricata | 1.414 | 1.111 | 1.155 | 1.318 | 1.324 |
| Eurya stenophylla | 2.29 | 0.766 | 0.835 | 1.618 | 1.63 |
| Eurya tsaii | 3.325 | 0.955 | 0.801 | 1.992 | 1.971 |
| Camellia melliana | 2.71 | 1.399 | 1.518 | 1.832 | 1.845 |
| Camellia transarisanensis | 2.83 | 0.87 | 0.947 | 1.821 | 1.835 |
| Eurya saxicola | 1.353 | 1.119 | 1.21 | 1.285 | 1.293 |
| Camellia cuspidata | 1.373 | 1.05 | 1.09 | 1.291 | 1.298 |
| Eurya macartneyi | 1.808 | 1.134 | 1.228 | 1.484 | 1.495 |
| Eurya patentipila | 2.557 | 0.929 | 1.034 | 1.74 | 1.753 |
| Ternstroemia nitida | 1.706 | 1.031 | 1.09 | 1.43 | 1.44 |
| Camellia euryoides | 1.883 | 1.083 | 1.126 | 1.514 | 1.521 |
| Cleyera lipingensis | 4.055 | 0.632 | 0.626 | 2.14 | 2.148 |
| Eurya rubiginosa | 1.401 | 1.312 | 1.379 | 1.329 | 1.336 |
| Adinandra millettii | 1.528 | 1.146 | 1.221 | 1.369 | 1.378 |
| Eurya hebeclados | 1.519 | 0.966 | 1.065 | 1.347 | 1.359 |
| Adinandra glischroloma | 2.042 | 0.945 | 1.05 | 1.554 | 1.567 |
| Eurya emarginata | 1.708 | 2.096 | 2.216 | 1.534 | 1.548 |
| Camellia drupifera | 2.343 | 0.829 | 1.022 | 1.644 | 1.663 |
| Adinandra nitida | 1.849 | 0.984 | 1.058 | 1.474 | 1.482 |
| Camellia polyodonta | 2.775 | 0.749 | 0.88 | 1.784 | 1.804 |
| Eurya acuminatissima | 1.92 | 1.009 | 1.11 | 1.519 | 1.531 |
| Eurya glandulosa | 1.963 | 1.245 | 1.299 | 1.547 | 1.552 |
| Schima wallichii | 2.791 | 0.899 | 0.796 | 1.811 | 1.803 |
| Pyrenaria spectabilis | 2.058 | 1.138 | 1.294 | 1.581 | 1.596 |
| Camellia cordifolia | 2.138 | 1.076 | 1.039 | 1.611 | 1.611 |
| Camellia petelotii | 2.903 | 0.752 | 0.897 | 1.826 | 1.85 |
| Eurya quinquelocularis | 3.767 | 0.84 | 0.722 | 2.084 | 2.072 |
| Eurya tetragonoclada | 2.514 | 0.876 | 0.841 | 1.72 | 1.721 |

| | | | | | |
|---|---|---|---|---|---|
| Pyrenaria hirta | 2.199 | 0.935 | 1.014 | 1.611 | 1.624 |
| Schima argentea | 2.214 | 0.837 | 0.786 | 1.606 | 1.604 |
| Stewartia villosa | 2.342 | 0.962 | 1.123 | 1.668 | 1.687 |
| Camellia semiserrata | 2.364 | 1.17 | 1.368 | 1.699 | 1.721 |
| Camellia costei | 2.329 | 0.889 | 0.865 | 1.657 | 1.66 |
| Camellia gymnogyna | 3.496 | 0.883 | 0.722 | 1.989 | 1.976 |
| Camellia crapnelliana | 1.852 | 1.292 | 1.436 | 1.522 | 1.537 |
| Camellia forrestii | 4.493 | 1.055 | 0.771 | 2.31 | 2.268 |
| Camellia reticulata | 2.958 | 0.987 | 0.818 | 1.874 | 1.856 |
| Camellia yunnanensis | 3.235 | 0.925 | 0.758 | 1.951 | 1.934 |
| Eurya pseudocerasifera | 3.468 | 0.913 | 0.834 | 2.025 | 2.009 |
| Cleyera pachyphylla | 1.759 | 0.922 | 1.025 | 1.44 | 1.452 |
| Eurya distichophylla | 2.253 | 1.162 | 1.229 | 1.656 | 1.665 |
| Adinandra bockiana | 2 | 0.863 | 0.915 | 1.537 | 1.546 |
| Cleyera incornuta | 3.245 | 0.737 | 0.714 | 1.932 | 1.933 |
| Stewartia pteropetiolata | 3.061 | 0.91 | 0.765 | 1.892 | 1.879 |
| Stewartia sinensis | 1.359 | 1.105 | 1.127 | 1.29 | 1.294 |
| Camellia taliensis | 3.514 | 0.882 | 0.738 | 2.028 | 2.012 |
| Camellia mairei | 2.861 | 0.921 | 0.819 | 1.83 | 1.822 |
| Schima brevipedicellata | 3.277 | 0.707 | 0.644 | 1.932 | 1.929 |
| Polyspora chrysandra | 3.406 | 0.887 | 0.745 | 1.997 | 1.982 |
| Adinandra hirta | 3.069 | 0.907 | 0.784 | 1.89 | 1.88 |
| Eurya jintungensis | 3.596 | 0.844 | 0.79 | 2.042 | 2.038 |
| Schima noronhae | 2.435 | 0.935 | 0.858 | 1.688 | 1.684 |
| Camellia saluenensis | 3.422 | 1 | 0.828 | 2.008 | 1.989 |
| Camellia brevistyla | 1.609 | 1.635 | 1.752 | 1.442 | 1.454 |
| Eurya cavinervis | 2.413 | 0.825 | 0.817 | 1.678 | 1.68 |
| Eurya obtusifolia | 2.901 | 0.851 | 0.804 | 1.834 | 1.834 |
| Camellia tsingpienensis | 5.292 | 0.903 | 0.754 | 2.44 | 2.425 |
| Eurya metcalfiana | 1.121 | 1.602 | 1.617 | 1.205 | 1.208 |

| | | | | | |
|---|---|---|---|---|---|
| Camellia salicifolia | 2.051 | 1.632 | 1.74 | 1.619 | 1.628 |
| Ternstroemia insignis | 4.277 | 0.898 | 0.822 | 2.231 | 2.228 |
| Eurya acuminoides | 2.659 | 0.801 | 0.82 | 1.756 | 1.763 |
| Eurya impressinervis | 2.728 | 0.815 | 0.835 | 1.781 | 1.787 |
| Eurya weissiae | 1.505 | 1.048 | 1.108 | 1.35 | 1.355 |
| Camellia rosthorniana | 2.727 | 0.786 | 0.781 | 1.779 | 1.784 |
| Camellia anlungensis | 3.744 | 1.131 | 0.895 | 2.113 | 2.083 |
| Eurya alata | 1.365 | 1.14 | 1.197 | 1.294 | 1.303 |
| Camellia tsaii | 3.691 | 0.984 | 0.811 | 2.08 | 2.06 |
| Camellia costata | 3.381 | 0.864 | 0.752 | 1.966 | 1.959 |
| Camellia crassicolumna | 5.335 | 1.122 | 0.792 | 2.524 | 2.473 |
| Eurya henryi | 3.666 | 1.104 | 0.783 | 2.059 | 2.024 |
| Eurya kueichowensis | 4.038 | 0.835 | 0.685 | 2.142 | 2.128 |
| Schima sinensis | 2.715 | 0.809 | 0.719 | 1.757 | 1.748 |
| Camellia tachangensis | 4.403 | 1.126 | 0.878 | 2.244 | 2.214 |
| Camellia pitardii | 3.158 | 0.81 | 0.69 | 1.903 | 1.89 |
| Eurya handel-mazzettii | 2.769 | 1.041 | 0.881 | 1.824 | 1.806 |
| Eurya oblonga | 2.607 | 0.979 | 0.893 | 1.731 | 1.725 |
| Polyspora longicarpa | 3.682 | 0.771 | 0.718 | 2.065 | 2.056 |
| Schima khasiana | 2.867 | 0.956 | 0.797 | 1.845 | 1.826 |
| Camellia grijsii | 1.743 | 1.171 | 1.22 | 1.466 | 1.474 |
| Polyspora speciosa | 2.884 | 0.835 | 0.79 | 1.822 | 1.822 |
| Camellia synaptica | 2.431 | 0.893 | 0.825 | 1.676 | 1.675 |
| Eurya fangii | 2.563 | 1.058 | 0.938 | 1.741 | 1.725 |
| Eurya pyracanthifolia | 2.94 | 1.008 | 0.875 | 1.866 | 1.854 |
| Camellia fraterna | 1.111 | 1.548 | 1.642 | 1.206 | 1.215 |
| Eurya brevistyla | 2.05 | 0.883 | 0.871 | 1.556 | 1.559 |
| Camellia chekiangoleosa | 1.245 | 1.586 | 1.65 | 1.279 | 1.286 |
| Eurya semiserrulata | 2.696 | 0.893 | 0.773 | 1.777 | 1.764 |
| Camellia rhytidocarpa | 4.237 | 0.486 | 0.531 | 2.166 | 2.178 |

| | | | | | |
|---|---|---|---|---|---|
| Camellia tuberculata | 3.324 | 0.811 | 0.77 | 1.964 | 1.958 |
| Camellia edithae | 3.916 | 1.519 | 1.544 | 2.225 | 2.229 |
| Eurya hupehensis | 4.81 | 0.625 | 0.693 | 2.314 | 2.333 |
| Camellia parvimuricata | 8.088 | 0.457 | 0.507 | 2.965 | 2.983 |
| Camellia lawii | 3.735 | 0.938 | 0.898 | 2.101 | 2.099 |
| Stewartia rostrata | 2.181 | 1.33 | 1.445 | 1.639 | 1.651 |
