# Supplementary material for: The Vulnerability of Chinese Theaceae Species Under Future Climate Change
Source: Biology (Basel). 2026 Jan 15;15(2):151. doi: 10.3390/biology15020151 (PMC12837319; doi:10.3390/biology15020151)
Supplement: Supplementary file 1 [file biology-15-00151-s001.zip › Table S2. The sensitivity factor of the 122 Chinese Theaceae species for four bioclimatic variables..pdf]

Table S2. The sensitivity factor of the 122 Chinese Theaceae species for four bioclimatic variables.

Speicies	BIO1	BIO7	BIO12	BIO15
Camellia caudata	5.765	5.499	2.757	4.289
Schima parviflora	3.233	2.893	2.543	1.893
Schima superba	3.765	3.164	2.617	2.112
Camellia furfuracea	5.514	4.875	1.853	4.553
Schima remotiserrata	6.307	6.543	3.267	3.400
Adinandra hainanensis	17.594	14.926	6.526	13.357
Camellia japonica	3.487	2.431	3.041	1.966
Camellia oleifera	3.428	2.886	2.880	1.609
Camellia sinensis	3.482	2.888	2.569	1.534
Eurya chinensis	4.152	4.199	3.020	3.480
Eurya ciliata	10.248	10.456	6.072	8.468
Eurya nitida	3.584	3.241	2.542	1.872
Eurya trichocarpa	10.410	9.304	5.351	6.013
Polyspora axillaris	8.867	6.198	2.860	7.929
Schima crenata	3.760	3.232	2.035	1.863
Ternstroemia kwangtungensis	3.863	3.734	2.237	2.559
Ternstroemia microphylla	16.804	16.162	5.322	10.282
Pyrenaria microcarpa	4.055	3.795	2.200	3.319
Anneslea fragrans	5.754	6.025	3.823	4.955
Cleyera japonica	2.355	2.075	1.575	1.267
Eurya groffii	7.062	6.312	3.965	4.880
Eurya japonica	2.459	1.539	2.142	1.131
Eurya loquaiana	3.201	2.909	2.454	1.936
Ternstroemia gymnanthera	3.339	3.106	2.259	2.240
Ternstroemia luteoflora	4.398	4.886	3.181	3.387
Camellia fluviatilis	13.965	14.316	6.228	7.471

Camellia kissii	9.384	7.897	5.225	6.498
Eurya acutisepala	7.459	6.248	4.574	3.612
Eurya muricata	2.673	1.504	2.317	1.508
Eurya stenophylla	6.482	7.251	3.688	3.563
Eurya tsaii	12.016	7.282	13.209	11.712
Camellia melliana	9.627	7.038	3.833	8.873
Camellia transarisanensis	10.026	7.696	8.293	6.027
Eurya saxicola	2.436	1.722	1.797	1.366
Camellia cuspidata	2.279	1.941	1.868	1.450
Eurya macartneyi	3.933	3.970	2.497	2.677
Eurya patentipila	7.286	8.644	5.096	5.130
Ternstroemia nitida	4.031	3.340	2.419	1.856
Camellia euryoides	4.352	3.677	2.719	3.432
Cleyera lipingensis	20.493	15.738	16.162	13.363
Eurya rubiginosa	2.690	1.703	1.928	1.531
Adinandra millettii	3.012	2.589	1.901	1.833
Eurya hebeclados	3.006	2.434	2.425	1.369
Adinandra glischroloma	5.386	4.946	3.452	2.901
Eurya emarginata	3.456	2.349	2.836	3.023
Camellia drupifera	5.991	6.723	3.811	5.436
Adinandra nitida	3.920	4.921	2.507	2.333
Camellia polyodonta	8.335	10.335	6.577	5.558
Eurya acuminatissima	4.148	4.331	3.180	3.083
Eurya glandulosa	4.259	4.571	2.105	4.480
Schima wallichii	10.290	7.518	6.852	6.498
Pyrenaria spectabilis	4.456	5.303	2.625	4.554
Camellia cordifolia	5.333	5.175	3.783	3.985
Camellia petelotii	11.849	8.856	8.226	4.775
Eurya quinquelocularis	13.556	20.771	12.733	9.693
Eurya tetragonoclada	7.155	7.457	5.630	5.048

Pyrenaria hirta	5.718	6.303	3.766	3.559
Schima argentea	6.396	5.059	3.736	4.417
Stewartia villosa	5.731	6.878	3.975	5.355
Camellia semiserrata	6.234	6.671	3.939	5.508
Camellia costei	6.400	5.839	5.015	4.441
Camellia gymnogyna	16.690	18.506	7.511	6.180
Camellia crapnelliana	4.195	3.738	2.572	3.219
Camellia forrestii	27.989	22.979	12.110	17.659
Camellia reticulata	11.673	7.029	10.029	6.260
Camellia yunnanensis	13.771	7.735	11.508	8.859
Eurya pseudocerasifera	14.940	9.182	10.412	13.579
Cleyera pachyphylla	4.190	3.565	2.397	2.218
Eurya distichophylla	6.599	5.567	3.291	4.848
Adinandra bockiana	4.813	4.110	4.089	2.994
Cleyera incornuta	12.656	12.786	8.549	8.140
Stewartia pteropetiolata	12.070	10.123	6.958	8.336
Stewartia sinensis	2.447	1.454	2.076	1.408
Camellia taliensis	16.082	11.569	9.916	11.834
Camellia mairei	9.603	10.039	7.296	5.794
Schima brevipedicellata	13.022	13.371	9.345	7.204
Polyspora chrysandra	13.937	10.818	13.207	8.446
Adinandra hirta	12.544	12.599	6.261	6.278
Eurya jintungensis	15.804	13.919	10.395	11.619
Schima noronhae	7.981	6.469	5.306	3.952
Camellia saluenensis	17.188	8.594	14.017	7.044
Camellia brevistyla	3.534	2.414	2.585	1.821
Eurya cavinervis	7.426	4.841	6.649	4.375
Eurya obtusifolia	11.228	9.411	7.506	5.528
Camellia tsingpienensis	41.696	41.254	13.281	15.801
Eurya metcalfiana	1.324	1.318	0.975	1.408

Camellia salicifolia	5.296	4.581	2.367	4.577
Ternstroemia insignis	23.894	21.552	15.041	12.695
Eurya acuminoides	7.840	9.009	6.471	4.959
Eurya impressinervis	9.570	8.544	5.491	6.155
Eurya weissiae	2.833	2.770	1.678	1.784
Camellia rosthorniana	8.868	8.010	6.738	6.135
Camellia anlungensis	19.142	15.778	11.318	9.845
Eurya alata	2.559	1.556	2.022	1.321
Camellia tsaii	17.726	14.431	13.184	9.141
Camellia costata	14.269	17.130	7.995	6.335
Camellia crassicolumna	37.573	29.104	14.917	32.249
Eurya henryi	19.570	18.165	10.023	5.992
Eurya kueichowensis	22.913	20.345	10.896	11.084
Schima sinensis	9.890	6.723	8.336	4.535
Camellia tachangensis	25.969	31.449	9.630	10.490
Camellia pitardii	12.978	10.644	9.936	6.334
Eurya handel-mazzettii	9.775	5.788	8.854	6.244
Eurya oblonga	9.265	8.949	5.585	3.382
Polyspora longicarpa	16.452	7.279	14.477	16.032
Schima khasiana	9.710	4.116	9.124	9.936
Camellia grijsii	4.067	2.210	3.859	2.013
Polyspora speciosa	8.722	12.357	7.315	4.874
Camellia synaptica	8.151	7.263	3.942	4.289
Eurya fangii	8.335	7.209	6.537	4.189
Eurya pyracanthifolia	11.905	7.530	9.734	5.416
Camellia fraterna	1.521	0.981	1.533	0.905
Eurya brevistyla	5.209	3.943	3.953	3.701
Camellia chekiangoleosa	1.923	1.307	1.762	1.207
Eurya semiserrulata	9.695	7.546	5.147	6.682
Camellia rhytidocarpa	18.297	17.660	19.476	16.382

Camellia tuberculata	12.249	10.665	10.171	11.121
Camellia edithae	16.139	21.163	10.663	13.371
Eurya hupehensis	36.474	22.627	13.128	20.326
Camellia parvimuricata	83.138	80.216	50.607	47.694
Camellia lawii	15.351	12.358	13.039	15.066
Stewartia rostrata	4.308	6.811	4.009	3.901
