# Supplementary material for: The Vulnerability of Chinese Theaceae Species Under Future Climate Change
Source: Biology (Basel). 2026 Jan 15;15(2):151. doi: 10.3390/biology15020151 (PMC12837319; doi:10.3390/biology15020151)
Supplement: Supplementary file 1 [file biology-15-00151-s001.zip › Table S3. The exposure factor of the 122 Chinese Theaceae species for four bioclimatic variables under RCP 2.6 scenario by the 2070s..pdf]

Table S3. The exposure factor of the 122 Chinese Theaceae species for four bioclimatic variables under RCP 2.6 scenario by the 2070s.

Speicies	BIO1	BIO7	BIO12	BIO15
Camellia caudata	0.347	0.202	0.781	0.593
Schima parviflora	0.446	0.265	0.749	0.657
Schima superba	0.459	0.294	1.165	0.714
Camellia furfuracea	0.403	0.245	0.836	0.709
Schima remotiserrata	0.326	0.357	0.700	0.603
Adinandra hainanensis	0.243	0.102	0.596	0.292
Camellia japonica	0.480	0.258	1.151	0.655
Camellia oleifera	0.402	0.233	0.714	0.657
Camellia sinensis	0.405	0.217	0.686	0.639
Eurya chinensis	0.432	0.223	0.795	0.659
Eurya ciliata	0.308	0.183	0.566	0.472
Eurya nitida	0.408	0.194	0.653	0.612
Eurya trichocarpa	0.281	0.164	0.563	0.494
Polyspora axillaris	0.406	0.310	1.493	0.748
Schima crenata	0.413	0.200	0.658	0.560
Ternstroemia kwangtungensis	0.393	0.222	0.756	0.516
Ternstroemia microphylla	0.210	0.106	0.598	0.281
Pyrenaria microcarpa	0.467	0.332	1.415	0.862
Anneslea fragrans	0.324	0.187	0.645	0.684
Cleyera japonica	0.484	0.259	0.780	0.656
Eurya groffii	0.339	0.185	0.583	0.665
Eurya japonica	0.658	0.233	0.992	0.735
Eurya loquaiana	0.396	0.238	0.649	0.656
Ternstroemia gymnanthera	0.375	0.226	0.672	0.679
Ternstroemia luteoflora	0.345	0.245	0.669	0.618
Camellia fluviatilis	0.240	0.135	0.534	0.393

Camellia kissii	0.321	0.149	0.611	0.537
Eurya acutisepala	0.294	0.242	0.494	0.526
Eurya muricata	0.488	0.277	0.755	0.591
Eurya stenophylla	0.286	0.202	0.495	0.468
Eurya tsaii	0.324	0.155	0.565	0.682
Camellia melliana	0.497	0.157	1.025	0.797
Camellia transarisanensis	0.265	0.215	0.696	0.395
Eurya saxicola	0.501	0.229	0.872	0.433
Camellia cuspidata	0.433	0.245	0.702	0.601
Eurya macartneyi	0.396	0.261	0.795	0.655
Eurya patentipila	0.318	0.269	0.603	0.569
Ternstroemia nitida	0.393	0.286	0.698	0.582
Camellia euryoides	0.391	0.298	0.768	0.586
Cleyera lipingensis	0.172	0.184	0.354	0.459
Eurya rubiginosa	0.573	0.272	0.897	0.716
Adinandra millettii	0.489	0.262	0.790	0.616
Eurya hebeclados	0.411	0.256	0.627	0.554
Adinandra glischroloma	0.348	0.220	0.636	0.565
Eurya emarginata	0.732	0.277	1.609	1.092
Camellia drupifera	0.284	0.146	0.663	0.381
Adinandra nitida	0.373	0.208	0.784	0.413
Camellia polyodonta	0.262	0.192	0.516	0.434
Eurya acuminatissima	0.382	0.226	0.688	0.590
Eurya glandulosa	0.442	0.189	0.905	0.706
Schima wallichii	0.298	0.189	0.453	0.691
Pyrenaria spectabilis	0.462	0.224	0.798	0.628
Camellia cordifolia	0.369	0.268	0.622	0.750
Camellia petelotii	0.355	0.200	0.429	0.464
Eurya quinquelocularis	0.251	0.196	0.453	0.633
Eurya tetragonoclada	0.296	0.234	0.485	0.625

Pyrenaria hirta	0.311	0.256	0.604	0.589
Schima argentea	0.264	0.203	0.473	0.606
Stewartia villosa	0.364	0.192	0.664	0.562
Camellia semiserrata	0.435	0.204	0.815	0.688
Camellia costei	0.282	0.225	0.502	0.640
Camellia gymnogyna	0.262	0.216	0.394	0.714
Camellia crapnelliana	0.536	0.291	0.945	0.637
Camellia forrestii	0.326	0.228	0.472	0.855
Camellia reticulata	0.327	0.190	0.533	0.739
Camellia yunnanensis	0.276	0.171	0.473	0.726
Eurya pseudocerasifera	0.291	0.151	0.557	0.645
Cleyera pachyphylla	0.333	0.284	0.681	0.442
Eurya distichophylla	0.411	0.215	0.774	0.732
Adinandra bockiana	0.341	0.240	0.564	0.503
Cleyera incornuta	0.261	0.235	0.416	0.497
Stewartia pteropetiolata	0.263	0.176	0.431	0.736
Stewartia sinensis	0.501	0.236	0.749	0.595
Camellia taliensis	0.236	0.190	0.461	0.688
Camellia mairei	0.301	0.213	0.396	0.745
Schima brevipedicellata	0.203	0.251	0.378	0.502
Polyspora chrysandra	0.276	0.172	0.480	0.671
Adinandra hirta	0.320	0.266	0.408	0.695
Eurya jintungensis	0.244	0.131	0.487	0.631
Schima noronhae	0.392	0.147	0.389	0.740
Camellia saluenensis	0.305	0.182	0.598	0.719
Camellia brevistyla	0.507	0.333	1.357	0.681
Eurya cavinervis	0.359	0.145	0.474	0.553
Eurya obtusifolia	0.287	0.192	0.459	0.628
Camellia tsingpienensis	0.276	0.180	0.376	0.752
Eurya metcalfiana	0.596	0.314	1.275	0.698

Camellia salicifolia	0.462	0.344	1.284	0.828
Ternstroemia insignis	0.224	0.222	0.517	0.663
Eurya acuminoides	0.301	0.213	0.464	0.539
Eurya impressinervis	0.277	0.242	0.527	0.500
Eurya weissiae	0.431	0.354	0.741	0.489
Camellia rosthorniana	0.260	0.207	0.434	0.565
Camellia anlungensis	0.331	0.231	0.407	0.975
Eurya alata	0.483	0.245	0.804	0.599
Camellia tsaii	0.269	0.180	0.443	0.817
Camellia costata	0.222	0.279	0.410	0.672
Camellia crassicolumna	0.302	0.234	0.335	1.001
Eurya henryi	0.339	0.212	0.383	0.955
Eurya kueichowensis	0.203	0.179	0.374	0.695
Schima sinensis	0.270	0.152	0.266	0.698
Camellia tachangensis	0.208	0.234	0.467	0.976
Camellia pitardii	0.259	0.169	0.369	0.652
Eurya handel-mazzettii	0.369	0.163	0.572	0.770
Eurya oblonga	0.309	0.181	0.361	0.837
Polyspora longicarpa	0.331	0.141	0.438	0.522
Schima khasiana	0.386	0.183	0.375	0.769
Camellia grijsii	0.556	0.241	0.831	0.561
Polyspora speciosa	0.295	0.231	0.388	0.638
Camellia synaptica	0.305	0.160	0.417	0.711
Eurya fangii	0.453	0.164	0.318	0.886
Eurya pyracanthifolia	0.338	0.166	0.564	0.746
Camellia fraterna	0.768	0.345	1.082	0.718
Eurya brevistyla	0.340	0.192	0.501	0.614
Camellia chekiangoleosa	0.730	0.339	1.134	0.763
Eurya semiserrulata	0.294	0.196	0.445	0.689
Camellia rhytidocarpa	0.161	0.135	0.297	0.322

Camellia tuberculata	0.244	0.171	0.312	0.687
Camellia edithae	0.613	0.192	1.101	0.826
Eurya hupehensis	0.217	0.169	0.386	0.407
Camellia parvimuricata	0.173	0.104	0.241	0.332
Camellia lawii	0.306	0.125	0.398	0.783
Stewartia rostrata	0.729	0.142	0.993	0.480
