# Supplementary material for: The Vulnerability of Chinese Theaceae Species Under Future Climate Change
Source: Biology (Basel). 2026 Jan 15;15(2):151. doi: 10.3390/biology15020151 (PMC12837319; doi:10.3390/biology15020151)
Supplement: Supplementary file 1 [file biology-15-00151-s001.zip › Table S4. The exposure factor of the 122 Chinese Theaceae species for four bioclimatic variables under RCP 8.5 scenario by the 2070s..pdf]

Table S4. The exposure factor of the 122 Chinese Theaceae species for four bioclimatic variables under RCP 8.5 scenario by the 2070s.

Speicies	BIO1	BIO7	BIO12	BIO15
Camellia caudata	0.466	0.200	0.906	0.523
Schima parviflora	0.610	0.272	0.829	0.606
Schima superba	0.594	0.294	1.278	0.671
Camellia furfuracea	0.543	0.251	0.961	0.647
Schima remotiserrata	0.459	0.376	0.828	0.559
Adinandra hainanensis	0.381	0.100	0.799	0.232
Camellia japonica	0.580	0.255	1.247	0.634
Camellia oleifera	0.519	0.235	0.758	0.594
Camellia sinensis	0.526	0.217	0.714	0.573
Eurya chinensis	0.581	0.227	0.930	0.594
Eurya ciliata	0.401	0.179	0.747	0.393
Eurya nitida	0.537	0.198	0.689	0.533
Eurya trichocarpa	0.392	0.162	0.719	0.414
Polyspora axillaris	0.457	0.299	1.633	0.659
Schima crenata	0.455	0.203	0.691	0.499
Ternstroemia kwangtungensis	0.554	0.221	0.870	0.428
Ternstroemia microphylla	0.329	0.105	0.797	0.229
Pyrenaria microcarpa	0.619	0.333	1.515	0.786
Anneslea fragrans	0.393	0.179	0.690	0.526
Cleyera japonica	0.607	0.267	0.820	0.597
Eurya groffii	0.439	0.179	0.651	0.549
Eurya japonica	0.792	0.233	0.972	0.687
Eurya loquaiana	0.515	0.239	0.653	0.571
Ternstroemia gymnanthera	0.472	0.223	0.665	0.569
Ternstroemia luteoflora	0.483	0.254	0.727	0.515
Camellia fluviatilis	0.363	0.130	0.723	0.366

Camellia kissii	0.422	0.149	0.764	0.455
Eurya acutisepala	0.401	0.248	0.540	0.424
Eurya muricata	0.629	0.282	0.738	0.560
Eurya stenophylla	0.387	0.216	0.575	0.413
Eurya tsaii	0.323	0.147	0.492	0.522
Camellia melliana	0.670	0.156	1.165	0.687
Camellia transarisanensis	0.388	0.220	0.757	0.353
Eurya saxicola	0.605	0.234	0.935	0.411
Camellia cuspidata	0.568	0.252	0.703	0.554
Eurya macartneyi	0.541	0.268	0.895	0.584
Eurya patentipila	0.403	0.281	0.769	0.487
Ternstroemia nitida	0.534	0.296	0.745	0.510
Camellia euryoides	0.541	0.305	0.805	0.484
Cleyera lipingensis	0.295	0.194	0.350	0.380
Eurya rubiginosa	0.704	0.274	0.952	0.652
Adinandra millettii	0.652	0.270	0.836	0.544
Eurya hebeclados	0.563	0.268	0.680	0.532
Adinandra glischroloma	0.473	0.227	0.772	0.481
Eurya emarginata	0.873	0.295	1.630	1.186
Camellia drupifera	0.447	0.157	0.848	0.319
Adinandra nitida	0.509	0.218	0.842	0.320
Camellia polyodonta	0.385	0.207	0.653	0.398
Eurya acuminatissima	0.507	0.230	0.799	0.531
Eurya glandulosa	0.574	0.194	0.994	0.575
Schima wallichii	0.353	0.170	0.458	0.519
Pyrenaria spectabilis	0.649	0.238	0.945	0.551
Camellia cordifolia	0.451	0.266	0.661	0.606
Camellia petelotii	0.521	0.202	0.535	0.454
Eurya quinquelocularis	0.278	0.186	0.441	0.463
Eurya tetragonoclada	0.392	0.234	0.490	0.509

Pyrenaria hirta	0.442	0.267	0.698	0.524
Schima argentea	0.336	0.195	0.481	0.486
Stewartia villosa	0.525	0.205	0.817	0.525
Camellia semiserrata	0.599	0.211	1.008	0.671
Camellia costei	0.407	0.227	0.466	0.561
Camellia gymnogyna	0.286	0.201	0.388	0.499
Camellia crapnelliana	0.719	0.302	1.060	0.576
Camellia forrestii	0.259	0.194	0.426	0.554
Camellia reticulata	0.350	0.162	0.479	0.540
Camellia yunnanensis	0.325	0.152	0.398	0.536
Eurya pseudocerasifera	0.288	0.127	0.646	0.423
Cleyera pachyphylla	0.426	0.299	0.786	0.403
Eurya distichophylla	0.557	0.220	0.884	0.609
Adinandra bockiana	0.481	0.251	0.596	0.434
Cleyera incornuta	0.318	0.240	0.439	0.398
Stewartia pteropetiolata	0.286	0.171	0.456	0.515
Stewartia sinensis	0.603	0.241	0.735	0.556
Camellia taliensis	0.276	0.168	0.486	0.452
Camellia mairei	0.332	0.202	0.391	0.605
Schima brevipedicellata	0.265	0.251	0.360	0.389
Polyspora chrysandra	0.296	0.159	0.456	0.483
Adinandra hirta	0.343	0.249	0.439	0.492
Eurya jintungensis	0.285	0.127	0.571	0.449
Schima noronhae	0.418	0.146	0.420	0.603
Camellia saluenensis	0.332	0.150	0.524	0.527
Camellia brevistyla	0.623	0.342	1.456	0.666
Eurya cavinervis	0.397	0.141	0.501	0.489
Eurya obtusifolia	0.358	0.198	0.436	0.538
Camellia tsingpienensis	0.286	0.174	0.373	0.563
Eurya metcalfiana	0.758	0.319	1.248	0.617

Camellia salicifolia	0.594	0.348	1.422	0.730
Ternstroemia insignis	0.287	0.225	0.523	0.519
Eurya acuminoides	0.391	0.229	0.427	0.533
Eurya impressinervis	0.388	0.247	0.574	0.395
Eurya weissiae	0.580	0.355	0.795	0.366
Camellia rosthorniana	0.355	0.212	0.428	0.507
Camellia anlungensis	0.381	0.196	0.219	0.755
Eurya alata	0.622	0.258	0.801	0.580
Camellia tsaii	0.227	0.163	0.444	0.618
Camellia costata	0.240	0.281	0.432	0.493
Camellia crassicolumna	0.252	0.205	0.258	0.675
Eurya henryi	0.283	0.178	0.309	0.637
Eurya kueichowensis	0.272	0.170	0.245	0.553
Schima sinensis	0.269	0.158	0.214	0.612
Camellia tachangensis	0.272	0.194	0.202	0.787
Camellia pitardii	0.289	0.159	0.287	0.534
Eurya handel-mazzettii	0.378	0.144	0.504	0.599
Eurya oblonga	0.358	0.178	0.276	0.749
Polyspora longicarpa	0.340	0.124	0.484	0.387
Schima khasiana	0.387	0.166	0.374	0.564
Camellia grijsii	0.653	0.257	0.838	0.542
Polyspora speciosa	0.360	0.245	0.360	0.552
Camellia synaptica	0.392	0.170	0.350	0.613
Eurya fangii	0.380	0.142	0.290	0.794
Eurya pyracanthifolia	0.363	0.150	0.529	0.575
Camellia fraterna	0.937	0.343	1.068	0.749
Eurya brevistyla	0.422	0.202	0.485	0.552
Camellia chekiangoleosa	0.880	0.337	1.087	0.809
Eurya semiserrulata	0.323	0.175	0.371	0.570
Camellia rhytidocarpa	0.270	0.140	0.353	0.254

Camellia tuberculata	0.341	0.179	0.178	0.643
Camellia edithae	0.828	0.206	1.145	0.586
Eurya hupehensis	0.316	0.185	0.440	0.390
Camellia parvimuricata	0.219	0.126	0.254	0.360
Camellia lawii	0.426	0.130	0.300	0.720
Stewartia rostrata	0.923	0.129	0.975	0.519
