# Supplementary material for: The Vulnerability of Chinese Theaceae Species Under Future Climate Change
Source: Biology (Basel). 2026 Jan 15;15(2):151. doi: 10.3390/biology15020151 (PMC12837319; doi:10.3390/biology15020151)
Supplement: Supplementary file 1 [file biology-15-00151-s001.zip › Table S5. The vulnerability factor of the 122 Chinese Theaceae species for four bioclimatic variables under RCP 2.6 scenario by the 2070s..pdf]

Table S5. The vulnerability factor of the 122 Chinese Theaceae species for four bioclimatic variables under RCP 2.6 scenario by the 2070s.

Speicies	BIO1	BIO7	BIO12	BIO15
Camellia caudata	2.787	2.571	2.216	2.614
Schima parviflora	2.162	1.913	2.109	1.771
Schima superba	2.344	2.024	2.380	1.903
Camellia furfuracea	2.781	2.464	1.845	2.790
Schima remotiserrata	2.892	2.980	2.356	2.334
Adinandra hainanensis	4.677	4.056	3.227	4.154
Camellia japonica	2.271	1.749	2.557	1.804
Camellia oleifera	2.192	1.886	2.221	1.633
Camellia sinensis	2.212	1.874	2.081	1.586
Eurya chinensis	2.439	2.266	2.328	2.403
Eurya ciliata	3.661	3.517	3.083	3.530
Eurya nitida	2.247	1.967	2.050	1.737
Eurya trichocarpa	3.652	3.290	2.892	2.997
Polyspora axillaris	3.530	2.850	2.670	3.723
Schima crenata	2.305	1.970	1.836	1.705
Ternstroemia kwangtungensis	2.320	2.137	1.982	1.970
Ternstroemia microphylla	4.510	4.228	2.916	3.629
Pyrenaria microcarpa	2.439	2.248	2.305	2.486
Anneslea fragrans	2.760	2.675	2.508	2.889
Cleyera japonica	1.869	1.616	1.675	1.449
Eurya groffii	3.075	2.735	2.506	2.851
Eurya japonica	2.019	1.378	2.066	1.401
Eurya loquaiana	2.114	1.898	2.011	1.790
Ternstroemia gymnanthera	2.143	1.952	1.943	1.939
Ternstroemia luteoflora	2.432	2.466	2.304	2.341
Camellia fluviatilis	4.162	4.031	3.091	3.226

Camellia kissii	3.521	3.012	2.901	3.160
Eurya acutisepala	3.107	2.786	2.614	2.347
Eurya muricata	1.994	1.386	2.017	1.549
Eurya stenophylla	2.887	2.952	2.348	2.287
Eurya tsaii	3.988	2.901	4.546	4.438
Camellia melliana	3.797	2.854	2.786	3.994
Camellia transarisanensis	3.561	3.058	3.751	2.900
Eurya saxicola	1.912	1.455	1.834	1.399
Camellia cuspidata	1.807	1.555	1.783	1.524
Eurya macartneyi	2.343	2.237	2.117	2.105
Eurya patentipila	3.099	3.312	2.859	2.838
Ternstroemia nitida	2.370	2.072	2.027	1.713
Camellia euryoides	2.460	2.184	2.192	2.333
Cleyera lipingensis	4.902	4.316	4.678	4.415
Eurya rubiginosa	2.057	1.472	1.913	1.621
Adinandra millettii	2.118	1.808	1.845	1.721
Eurya hebeclados	2.059	1.748	1.986	1.459
Adinandra glischroloma	2.695	2.456	2.376	2.131
Eurya emarginata	2.446	1.732	2.720	2.515
Camellia drupifera	2.774	2.776	2.518	2.740
Adinandra nitida	2.320	2.438	2.115	1.816
Camellia polyodonta	3.243	3.511	3.158	2.823
Eurya acuminatissima	2.394	2.304	2.317	2.214
Eurya glandulosa	2.479	2.331	2.003	2.764
Schima wallichii	3.655	2.990	3.156	3.315
Pyrenaria spectabilis	2.552	2.548	2.172	2.723
Camellia cordifolia	2.702	2.561	2.477	2.641
Camellia petelotii	4.008	3.259	3.428	2.644
Eurya quinquelocularis	4.117	4.984	4.300	3.978
Eurya tetragonoclada	3.045	3.033	2.891	2.864

Pyrenaria hirta	2.738	2.813	2.458	2.378
Schima argentea	2.843	2.467	2.345	2.663
Stewartia villosa	2.796	2.864	2.572	2.892
Camellia semiserrata	2.991	2.834	2.674	3.050
Camellia costei	2.864	2.675	2.744	2.699
Camellia gymnogyna	4.589	4.744	3.236	3.255
Camellia crapnelliana	2.538	2.196	2.236	2.295
Camellia forrestii	6.093	5.313	4.223	5.724
Camellia reticulata	3.936	2.892	3.921	3.300
Camellia yunnanensis	4.192	3.009	4.117	3.910
Eurya pseudocerasifera	4.392	3.251	4.027	4.727
Cleyera pachyphylla	2.364	2.139	2.007	1.789
Eurya distichophylla	3.052	2.600	2.417	2.898
Adinandra bockiana	2.541	2.257	2.529	2.122
Cleyera incornuta	3.995	3.973	3.479	3.490
Stewartia pteropetiolata	3.904	3.451	3.155	3.805
Stewartia sinensis	1.917	1.341	1.906	1.498
Camellia taliensis	4.458	3.710	3.806	4.470
Camellia mairei	3.535	3.489	3.191	3.180
Schima brevipedicellata	3.958	4.089	3.589	3.290
Polyspora chrysandra	4.217	3.561	4.422	3.756
Adinandra hirta	4.070	3.993	2.969	3.262
Eurya jintungensis	4.434	3.968	3.931	4.354
Schima noronhae	3.333	2.724	2.714	2.623
Camellia saluenensis	4.737	3.187	4.732	3.480
Camellia brevistyla	2.308	1.794	2.469	1.749
Eurya cavinervis	3.177	2.354	3.131	2.606
Eurya obtusifolia	3.801	3.349	3.309	3.000
Camellia tsingpienensis	7.295	6.976	4.275	5.261
Eurya metcalfiana	1.454	1.316	1.489	1.546

Camellia salicifolia	2.782	2.481	2.325	2.892
Ternstroemia insignis	5.409	5.132	4.776	4.595
Eurya acuminoides	3.194	3.306	3.078	2.763
Eurya impressinervis	3.496	3.258	2.896	3.039
Eurya weissiae	2.013	1.936	1.709	1.630
Camellia rosthorniana	3.343	3.109	3.108	3.098
Camellia anlungensis	5.047	4.407	3.990	4.410
Eurya alata	1.948	1.392	1.910	1.454
Camellia tsaii	4.742	4.126	4.362	4.075
Camellia costata	4.175	4.681	3.357	3.255
Camellia crassicolumna	6.993	5.993	4.462	8.032
Eurya henryi	5.118	4.693	3.724	3.422
Eurya kueichowensis	5.251	4.898	3.869	4.335
Schima sinensis	3.544	2.783	3.249	2.775
Camellia tachangensis	5.601	6.230	3.758	4.553
Camellia pitardii	4.043	3.527	3.688	3.235
Eurya handel-mazzettii	3.658	2.594	3.731	3.325
Eurya oblonga	3.483	3.250	2.757	2.492
Polyspora longicarpa	4.679	2.882	4.563	4.940
Schima khasiana	3.668	2.207	3.542	4.193
Camellia grijsii	2.516	1.656	2.658	1.773
Polyspora speciosa	3.360	3.900	3.186	2.826
Camellia synaptica	3.261	2.902	2.364	2.709
Eurya fangii	3.480	2.897	2.935	2.811
Eurya pyracanthifolia	3.991	2.963	3.902	3.075
Camellia fraterna	1.640	1.149	1.786	1.247
Eurya brevistyla	2.642	2.168	2.436	2.444
Camellia chekiangoleosa	1.824	1.323	1.939	1.458
Eurya semiserrulata	3.541	3.004	2.727	3.360
Camellia rhytidocarpa	4.609	4.477	5.026	4.653

Camellia tuberculata	3.903	3.534	3.653	4.331
Camellia edithae	5.102	5.024	4.734	4.941
Eurya hupehensis	6.664	5.142	4.266	5.348
Camellia parvimuricata	9.874	9.408	7.923	7.969
Camellia lawii	4.477	3.729	4.269	5.183
Stewartia rostrata	2.729	2.789	2.827	2.403
