# Supplementary material for: The Vulnerability of Chinese Theaceae Species Under Future Climate Change
Source: Biology (Basel). 2026 Jan 15;15(2):151. doi: 10.3390/biology15020151 (PMC12837319; doi:10.3390/biology15020151)
Supplement: Supplementary file 1 [file biology-15-00151-s001.zip › Table S6. The vulnerability factor of the 122 Chinese Theaceae species for four bioclimatic variables under RCP 8.5 scenario by the 2070s..pdf]

Table S6. The vulnerability factor of the 122 Chinese Theaceae species for four bioclimatic variables under RCP 8.5 scenario by the 2070s.

Speicies	BIO1	BIO7	BIO12	BIO15
Camellia caudata	2.907	2.569	2.292	2.555
Schima parviflora	2.281	1.919	2.156	1.744
Schima superba	2.450	2.024	2.442	1.879
Camellia furfuracea	2.917	2.469	1.906	2.739
Schima remotiserrata	3.034	3.001	2.444	2.302
Adinandra hainanensis	4.929	4.052	3.426	4.056
Camellia japonica	2.347	1.747	2.614	1.793
Camellia oleifera	2.282	1.888	2.250	1.601
Camellia sinensis	2.305	1.875	2.098	1.554
Eurya chinensis	2.562	2.270	2.415	2.355
Eurya ciliata	3.789	3.511	3.257	3.434
Eurya nitida	2.347	1.971	2.072	1.694
Eurya trichocarpa	3.807	3.287	3.033	2.915
Polyspora axillaris	3.594	2.838	2.744	3.626
Schima crenata	2.339	1.971	1.855	1.671
Ternstroemia kwangtungensis	2.450	2.135	2.045	1.912
Ternstroemia microphylla	4.726	4.227	3.092	3.555
Pyrenaria microcarpa	2.562	2.250	2.352	2.435
Anneslea fragrans	2.831	2.665	2.542	2.750
Cleyera japonica	1.945	1.622	1.693	1.423
Eurya groffii	3.188	2.728	2.558	2.749
Eurya japonica	2.099	1.378	2.055	1.381
Eurya loquaiana	2.202	1.898	2.014	1.744
Ternstroemia gymnanthera	2.217	1.949	1.940	1.875
Ternstroemia luteoflora	2.554	2.475	2.344	2.265
Camellia fluviatilis	4.364	4.021	3.275	3.194

Camellia kissii	3.653	3.012	3.036	3.075
Eurya acutisepala	3.232	2.793	2.654	2.267
Eurya muricata	2.087	1.389	2.007	1.534
Eurya stenophylla	2.999	2.969	2.410	2.244
Eurya tsaii	3.987	2.891	4.440	4.222
Camellia melliana	4.010	2.853	2.881	3.869
Camellia transarisanensis	3.731	3.064	3.817	2.856
Eurya saxicola	1.977	1.458	1.865	1.389
Camellia cuspidata	1.890	1.559	1.784	1.501
Eurya macartneyi	2.462	2.244	2.176	2.059
Eurya patentipila	3.197	3.327	3.003	2.762
Ternstroemia nitida	2.487	2.081	2.055	1.674
Camellia euryoides	2.590	2.190	2.215	2.257
Cleyera lipingensis	5.152	4.335	4.671	4.295
Eurya rubiginosa	2.141	1.473	1.940	1.590
Adinandra millettii	2.230	1.813	1.868	1.682
Eurya hebeclados	2.167	1.757	2.019	1.448
Adinandra glischroloma	2.817	2.463	2.473	2.073
Eurya emarginata	2.544	1.744	2.731	2.571
Camellia drupifera	2.944	2.789	2.654	2.678
Adinandra nitida	2.432	2.448	2.149	1.755
Camellia polyodonta	3.397	3.532	3.297	2.787
Eurya acuminatissima	2.500	2.308	2.392	2.173
Eurya glandulosa	2.589	2.336	2.049	2.657
Schima wallichii	3.731	2.966	3.161	3.142
Pyrenaria spectabilis	2.711	2.562	2.260	2.658
Camellia cordifolia	2.782	2.559	2.507	2.530
Camellia petelotii	4.246	3.262	3.553	2.635
Eurya quinquelocularis	4.162	4.964	4.283	3.766
Eurya tetragonoclada	3.156	3.033	2.897	2.760

Pyrenaria hirta	2.871	2.826	2.529	2.329
Schima argentea	2.923	2.458	2.352	2.562
Stewartia villosa	2.957	2.879	2.687	2.858
Camellia semiserrata	3.157	2.843	2.813	3.034
Camellia costei	3.000	2.677	2.711	2.633
Camellia gymnogyna	4.633	4.714	3.229	3.044
Camellia crapnelliana	2.685	2.206	2.302	2.252
Camellia forrestii	5.936	5.239	4.156	5.239
Camellia reticulata	3.970	2.858	3.851	3.104
Camellia yunnanensis	4.272	2.986	4.011	3.689
Eurya pseudocerasifera	4.386	3.217	4.139	4.396
Cleyera pachyphylla	2.445	2.152	2.069	1.764
Eurya distichophylla	3.206	2.607	2.490	2.793
Adinandra bockiana	2.670	2.267	2.555	2.072
Cleyera incornuta	4.085	3.982	3.507	3.374
Stewartia pteropetiolata	3.940	3.444	3.183	3.554
Stewartia sinensis	1.981	1.343	1.898	1.480
Camellia taliensis	4.530	3.676	3.839	4.146
Camellia mairei	3.577	3.473	3.185	3.050
Schima brevipedicellata	4.059	4.090	3.565	3.164
Polyspora chrysandra	4.250	3.541	4.386	3.539
Adinandra hirta	4.104	3.967	3.002	3.060
Eurya jintungensis	4.506	3.960	4.042	4.103
Schima noronhae	3.364	2.723	2.745	2.517
Camellia saluenensis	4.785	3.144	4.622	3.280
Camellia brevistyla	2.395	1.800	2.520	1.742
Eurya cavinervis	3.221	2.350	3.159	2.552
Eurya obtusifolia	3.904	3.358	3.282	2.916
Camellia tsingpienensis	7.322	6.961	4.270	4.970
Eurya metcalfiana	1.526	1.319	1.480	1.509

Camellia salicifolia	2.905	2.485	2.394	2.813
Ternstroemia insignis	5.545	5.137	4.787	4.392
Eurya acuminoides	3.302	3.328	3.039	2.757
Eurya impressinervis	3.645	3.264	2.939	2.930
Eurya weissiae	2.115	1.937	1.735	1.561
Camellia rosthorniana	3.466	3.116	3.102	3.040
Camellia anlungensis	5.141	4.345	3.715	4.156
Eurya alata	2.037	1.399	1.908	1.445
Camellia tsaii	4.665	4.096	4.363	3.846
Camellia costata	4.206	4.684	3.384	3.075
Camellia crassicolumna	6.859	5.921	4.332	7.350
Eurya henryi	5.011	4.626	3.623	3.132
Eurya kueichowensis	5.398	4.879	3.683	4.149
Schima sinensis	3.543	2.790	3.181	2.704
Camellia tachangensis	5.747	6.129	3.403	4.329
Camellia pitardii	4.090	3.512	3.575	3.117
Eurya handel-mazzettii	3.671	2.573	3.649	3.160
Eurya oblonga	3.547	3.247	2.670	2.432
Polyspora longicarpa	4.695	2.861	4.635	4.715
Schima khasiana	3.670	2.191	3.540	3.942
Camellia grijsii	2.593	1.667	2.664	1.762
Polyspora speciosa	3.444	3.923	3.154	2.751
Camellia synaptica	3.368	2.915	2.307	2.630
Eurya fangii	3.391	2.869	2.904	2.742
Eurya pyracanthifolia	4.029	2.942	3.858	2.921
Camellia fraterna	1.717	1.148	1.780	1.258
Eurya brevistyla	2.721	2.177	2.423	2.397
Camellia chekiangoleosa	1.901	1.322	1.918	1.477
Eurya semiserrulata	3.581	2.978	2.657	3.238
Camellia rhytidocarpa	4.820	4.488	5.133	4.533

Camellia tuberculata	4.052	3.545	3.461	4.274
Camellia edithae	5.432	5.051	4.783	4.606
Eurya hupehensis	6.929	5.179	4.348	5.316
Camellia parvimuricata	10.066	9.505	7.965	8.053
Camellia lawii	4.679	3.737	4.117	5.091
Stewartia rostrata	2.878	2.772	2.814	2.434
